# Assessment of Optimal Conditions for Marine Invertebrate Cell-Mediated Mineralization of Organic Matrices

**DOI:** 10.3390/biomimetics7030086

**Published:** 2022-06-26

**Authors:** Jeremy Elias, Thomas Angelini, Mark Q. Martindale, Laurie Gower

**Affiliations:** 1 Department of Materials Science & Engineering, University of Florida, Gainesville, FL 32611, USA; jelias@ufl.edu; 2 Department of Mechanical and Aerospace Engineering, University of Florida, Gainesville, FL 32611, USA; t.e.angelini@ufl.edu; 3 Whitney Laboratory of Marine Bioscience, University of Florida, St. Augustine, FL 32080, USA; mqmartin@whitney.ufl.edu

**Keywords:** biomineralization, biomimetic processing, PILP, collagen mineralization

## Abstract

Cellular strategies and regulation of their crystallization mechanisms are essential to the formation of biominerals, and harnessing these strategies will be important for the future creation of novel non-native biominerals that recapitulate the impressive properties biominerals possess. Harnessing these biosynthetic strategies requires an understanding of the interplay between insoluble organic matrices, mineral precursors, and soluble organic and inorganic additives. Our long-range goal is to use a sea anemone model system (*Nematostella vectensis*) to examine the role of intrinsically disordered proteins (IDPs) found in native biomineral systems. Here, we study how ambient temperatures (25–37 °C) and seawater solution compositions (varying NaCl and Mg ratios) will affect the infiltration of organic matrices with calcium carbonate mineral precursors generated through a polymer-induced liquid-precursor (PILP) process. Fibrillar collagen matrices were used to assess whether solution conditions were suitable for intrafibrillar mineralization, and SEM with EDS was used to analyze mineral infiltration. Conditions of temperatures 30 °C and above and with low Mg:Ca ratios were determined to be suitable conditions for calcium carbonate infiltration. The information obtained from these observations may be useful for the manipulation and study of cellular secreted IDPs in our quest to create novel biosynthetic materials.

## 1. Introduction

Complex cellular processes provide control over many crystallographic features in biomineralization, such as crystal size, orientation, phase, composition, texture, and crystal location. These processes control the assembly of nanoscale components into hierarchically structured composite materials. This control over crystal nucleation and growth creates structures with remarkable properties, such as combinations of high strength and toughness [1,2,3,4] and unique photonic and magnetic properties [5,6,7]. Biomineralization processes are generally restricted to materials and structures found in physiological environments, but manipulating and guiding cellular processes provides a pathway to a wider variety of biosynthetic processing, including the potential for synthesis with non-native materials. Toward this grand challenge, our group’s recent research has focused on developing a better understanding of how to harness these cellular strategies. This is being examined by genetically programming cells from non-mineralizing organisms, such as cnidarians (sea anemones, *Nematostella vectensis*), to secrete non-native proteins to direct the process of biomineralization of organic matrices provided to them in the surrounding mineral salt-containing solutions. In order to successfully achieve this goal, an understanding of the optimal conditions for both cell health and matrix infiltration must be reached.

Cells regulate the biomineralization process by first secreting insoluble structural biopolymers (e.g., collagen and chitin) that have long been understood to serve as a self-organizing scaffold that directs mineral nucleation and growth [8,9]. These biopolymers are recognized for their role in templating and patterning processes such as calcification [10,11] and silicification [12,13]. Many of the matrices also play a role in guiding the formation of interpenetrating organic-mineral composites by providing nanoscale infiltration compartments [14,15]. In addition to these insoluble matrix components, cells also secrete soluble proteins that interact with mineral precursors, such as prenucleation clusters [16,17] and liquid-condensed phases [18,19], enabling them to accumulate into metastable amorphous phases that can be molded and shaped to form species-specific, non-equilibrium morphologies via a non-classical crystallization pathway [20,21]. These soluble proteins are highly charged and therefore tend not to fold into globular structures and instead fall in the category of intrinsically disordered proteins (IDPs) [22,23,24,25]. In materials such as bone, mineralization of the collagen matrix is facilitated by non-collagenous proteins (NCPs), most of which are IDPs [22,26,27]. Current evidence from in vitro model systems, such as the polymer-induced liquid-precursor (PILP) process discovered in our lab [21,28], suggests the NCPs help to stabilize amorphous precursor phases that have fluidic character and are able to infiltrate the interstices of collagen matrices [10,29], where the amorphous phase then crystallizes within the narrow confines of the fibrillar scaffold to template the oriented nanocrystals of hydroxyapatite [10,29]. Similar organic-mineral interactions contribute to the hierarchical structure of invertebrate exoskeletons that use chitin as their main insoluble matrix component. In structures such as crustacean cuticle and nacre, many acidic macromolecules (the soluble IDPs) have been identified and are considered to be crucial in modulating the calcification process [30], such as by stabilizing amorphous calcium carbonate and phosphate phases within the crustacean cuticle structures [31,32] or providing control over the final crystalline phases of calcium carbonate in nacre [33].

An understanding of the interplay between these soluble proteins, mineralizing solution conditions, and insoluble organic matrices is needed before we can begin to manipulate cell-secreted biomineralization. To emulate the role of the IDPs we plan to study in the future, poly(acrylic acid) (PAA) was used here to induce the polymer-induced liquid-precursor (PILP) process, which has been hypothesized as being one of the primary roles of the IDPs in modulating biomineralization [20,21]. PAA is a simple and inexpensive mimic for the acidic proteins found in biomineralizing organisms and has been shown in vitro to generate amorphous mineral precursors that are able to be molded and shaped into non-equilibrium morphologies [34,35], creating mineral structures through processes that appear similar to those observed in natural biomineral formation [36,37]. For the insoluble matrix component, type I collagen was used because it provides a convenient system for the study of non-classical mineralization processes (such as the PILP process), where the fibrils display a distinctly different appearance upon intrafibrillar mineralization. When viewed by scanning electron microscopy (SEM), the fibrils exhibit a less smooth texture, and they remain ‘plump’ in regions where water has been replaced with minerals [29,38]. Fibrillar collagen systems have been used to analyze the infiltration of calcium carbonate [38,39], silica [40], and iron oxide [10] mineral precursors, in addition to calcium phosphate for the study of bone formation [10,29,41]. However, even though collagen is found in invertebrate organisms, it does not appear to be a part of their biomineralized structures, as invertebrate biominerals in organisms such as arthropods and mollusks are often chitin-based [42]. Because of this, we were curious about the effect of marine conditions on collagen mineralization.

In contrast to the beneficial role of inorganic species such as Mg in invertebrate biominerals such as crustacean exoskeletons, where they serve to stabilize amorphous phases that are essential to skeletal formation and structure, these ions seem to play a different role with respect to vertebrate biominerals. Magnesium is known to be essential to bone formation and physiological properties, and Mg deficiency leads to poor bone formation in vertebrates [43]. However, unlike invertebrate structures, Mg is not incorporated into bone structures in high amounts (less than 1%) [44], and in vitro studies of cell-directed collagen matrix deposition and mineralization have shown that excessive Mg can disrupt the effective mineral infiltration of collagen [43]. Therefore, solution conditions mimicking the concentration of seawater were chosen here as the starting point for this work, both to accommodate the viability of marine invertebrate cells for the future studies on the secretion of biomineralization IDPs and to investigate the effect of seawater Mg/Ca ratios and Ca concentrations on the infiltration of collagen matrices with CaCO_3_.

Given that vertebrates and invertebrates operate at different physiological temperatures, we were also interested in determining the temperature range for which these reaction conditions would be effective. Mineralization studies in collagen systems are typically carried out at mammalian physiological temperatures of 37 °C, which is considerably higher than the temperatures of marine invertebrates, which generally develop at ambient temperatures and are therefore lower than 30 °C [45,46]. Therefore, an understanding of temperature effects may be significant for the analysis and control of these cellular processes in addition to the solution compositions described above. In the current work, this complex interplay between temperature and solution conditions was examined through the mineralization of type I collagen matrices in the presence of soluble process-directing agent (PAA) within artificial seawater (ASW) solutions containing variable ratios of inorganics, and over a range of temperatures (24–37 °C), with an assessment of effective mineral infiltration through SEM/EDS and XRD analyses.

## 2. Materials and Methods

### 2.1. Collagen Preparation

Collagen scaffolds were prepared from concentrated type I collagen solutions (TeloCol^®^, Advanced Biomatrix, Carlsbad, CA, USA) at 3 mg/mL. The collagen was neutralized through the addition of 0.1 M NaOH, and solutions were deposited onto glass coverslips (Fisher Scientific, Hampton, NH, USA). Collagen-coated coverslips were incubated at 37 °C for 1 h to induce collagen gelation and fibrillogenesis.

### 2.2. Collagen Mineralization

Calcium carbonate solutions were prepared by the addition of calcium chloride (CaCl_2_•2H_2_O, Fisher Scientific, Hampton, NH, USA), magnesium chloride (MgCl_2_•6H_2_O, Fisher, Hampton, NH, USA), and sodium chloride (NaCl, Fisher, Hampton, NH, USA), or an artificial seawater mix (Instant Ocean, Blacksburg, VA, USA) to DI H_2_O. Four different solution conditions were created, with compositions shown in Table 1. Poly(acrylic acid, sodium salt) (PAA, 15 kDa, Sigma-Aldrich, St. Louis, MO, USA) was added to a concentration of 20 µg/mL through the addition of aliquots from a PAA stock solution (35 wt%) to the mineralizing solutions, to serve as the process-directing agent. Collagen-coated coverslips were then immersed in each solution in plastic Petri dishes (35 mm diameter × 10 mm height, Fisher Scientific, Hampton, NH, USA), and mineralization was carried out through the vapor diffusion method. Ammonium carbonate powder (NH_4_CO_3_, ACROS organics, Hampton, NH, USA) was crushed and placed into vial caps that were sealed with parafilm. The parafilm was punctured with a needle to allow the vapor to escape, and the mineralization solutions were incubated at either room temperature, 30 °C, or 37 °C, in the presence of the ammonium carbonate vapors (CO_2_ and NH_3_) produced by the decomposition of the NH_4_CO_3_, which then diffuses throughout the sealed desiccator and into the mineralizing dishes. The reaction was allowed to incubate for 3 days, and the collagen-coated coverslips were then rinsed with water and dried under ambient conditions.

### 2.3. pH Analysis

To examine the possible effects of solution pH on successful mineralization of scaffolds, pH was measured before mineralization and each day after samples were incubated in mineralization solution. pH was measured by a Fisherbrand™ Accumet™ AB200 pH meter.

### 2.4. Scanning Electron Microscopy (SEM) and Energy Dispersive Spectroscopic (EDS) Analysis

Collagen-coated coverslips were rinsed 3 times with DI H_2_O and air-dried after mineralization, and each slide was mounted onto an SEM stub and carbon-coated for analysis. Each scaffold was imaged in a TESCAN MIRA3 SEM equipped with an EDAX Octane Pro SDD energy dispersive spectrometer (EDS) at an accelerating voltage of 5 kV. To characterize chemical composition and confirm the presence of calcium carbonate minerals, EDS was performed at an accelerating voltage of 15 kV.

### 2.5. X-ray Diffraction (XRD) Analysis

X-Ray powder diffraction patterns were collected using a Rigaku Miniflex X-Ray diffractometer using CuKα radiation at 40 kV and 15 mA. The diffraction pattern was collected at a scanning rate of 0.2 degrees per second with a diffraction angle of 2θ ranging from 5 to 70°.

## 3. Results

### 3.1. Mineralization at Room Temperature

The starting concentration for the artificial seawater (ASW) solutions was chosen at ½ the concentration of standard seawater (Table 1), and the initial temperatures were the laboratory ambient temperature of ~24 °C. The diluted seawater was used to provide milder concentrations that were closer to the diluted seawater compositions used by collaborators at the Whitney Laboratory for Marine Bioscience to dissociate cnidarian embryos, which would be used in our next set of studies with individual cnidarian cells programmed to secrete IDPs into the reaction media.

**Figure 1:**
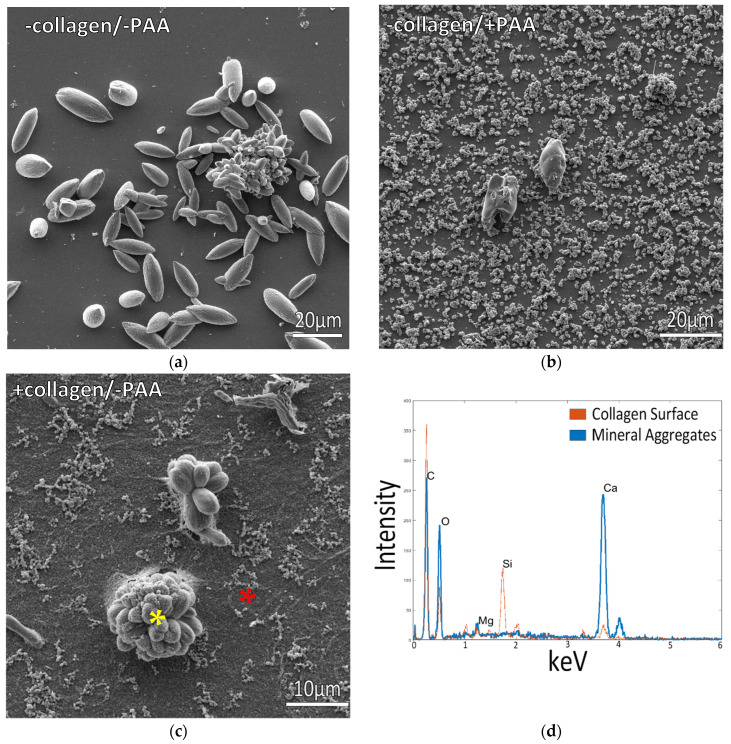
Glass slides incubated in ½ ASW solutions at room temperature. (**a**) Uncoated collagen slides incubated in solutions containing no polymer displayed large spindle-shaped mineral aggregates on the surface. (**b**) Solutions containing 20 μg/mL PAA displayed smaller, more granular precipitates, with only a few of the large aggregates. (**c**) Collagen-coated glass slides under the same conditions as (**a**) displayed bundles of mineral aggregates on the surface. (**d**) EDS measurements from sample (**c**) are shown as the blue spectrum for the large mineral aggregate (marked by the yellow asterisk), with the red spectrum taken from the background region (red asterisk), indicating low mineral deposition on the surrounding collagen surface.

For the initial control reactions, glass coverslips with no collagen coating were examined. They displayed mineral morphologies that were expected of their respective solution compositions, with lens or spindle-shaped bundles forming in mineralizing solutions without polymer (Figure 1a), typical of Mg modified calcite [47], as verified by XRD (Supplementary Figure S1a). Adding PAA to the solutions resulted in smaller mineral aggregates that displayed much smoother textures amidst a large collection of tiny granular precipitates, roughly a micron in size (Figure 1b).

**Figure 2:**
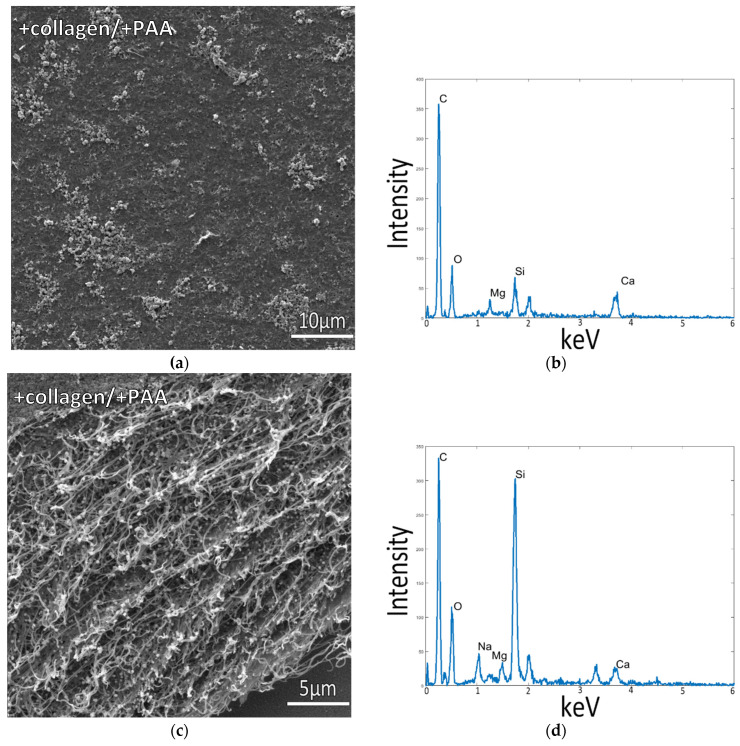
Collagen-coated glass slide incubated in 1/2x ASW with PAA in solution at room temperature. SEM image in (**a**) displays the flat collagen surface, and (**c**) shows a cross-section of the cut collagen surface, where the flat appearance of the collagen fibrils and lack of mineral precipitates indicates poor mineral infiltration into the collagen matrix. EDS spectra in (**b**,**d**), taken of samples shown in (**a**,**c**), respectively, reveal a low Ca signal, further indicating a low level of mineral infiltration into the matrix.

When using collagen-coated coverslips, incubation in ASW at ½ concentration without any polymer additive resulted in the deposition of large mineral agglomerates on the collagen surface, while the collagen matrix displayed a flattened texture, indicating poor mineral infiltration into the fibrils of the matrix (Figure 1c,d). Adding PAA to these ½ ASW solutions resulted in reduced mineral deposition with fewer large aggregates on the collagen surface (Figure 2a), and the collagen film underneath the mineral remained poorly mineralized (Figure 2c,d). However, the collagen surfaces in both Figure 1c and Figure 2 appear to be decorated with a few strands and clumps of small particulates, and upon closer inspection, similar submicron particles appear to have formed a fine-grained coating that shrouds the fibrillar texture of the collagen. In Figure 2c, this particulate coating can be seen at the edge of the upper surface, while some particulates also appear to be scattered throughout the interior of the cross-section. These fine particulates are likely responsible for the small calcium signal in the EDS spectra of these collagen matrices. XRD analysis of the room temperature samples indicated that the precipitates from ½ ASW + PAA solutions, on both the glass and collagen substrates, were a mixture of calcite and aragonite crystals (Supplementary Figure S1b,c). The peak widths are substantially broader than the reference samples, presumably due to the small size of the crystallites.

**Figure 3:**
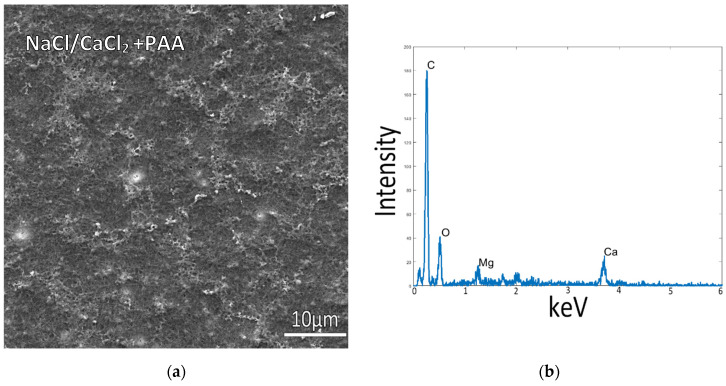
Collagen-coated glass slide incubated at 150 mM NaCl + 10 mM CaCl_2_ concentrations with PAA in solution, at room temperature. (**a**) The flat collagen surface and lack of precipitates, as well as the low calcium signal in the EDS spectrum (**b**), indicate relatively low mineral deposition on the collagen surface or within the fibrils.

To examine mineralization behavior in the absence of non-calcium mineral additives (namely Mg ions, which are known to have a pronounced influence on CaCO_3_ precipitation), simplified solutions were created to remove the Mg ions. Samples incubated at room temperature in a solution of only NaCl and 10 mM CaCl_2_ (without Mg) showed similar mineralization, with tiny and few mineral particles on top of poorly mineralized collagen (Figure 3a). In these samples, the fibrillar texture was more easily visible, but the collagen matrices were still poorly mineralized, as indicated by the EDS spectrum (Figure 3b). This sample had a weak XRD signal due to the small amount of mineral, so only a few broad peaks of calcite were detected (Supplementary Figure S1d).

### 3.2. Mineralization at 37 °C

**Figure 4:**
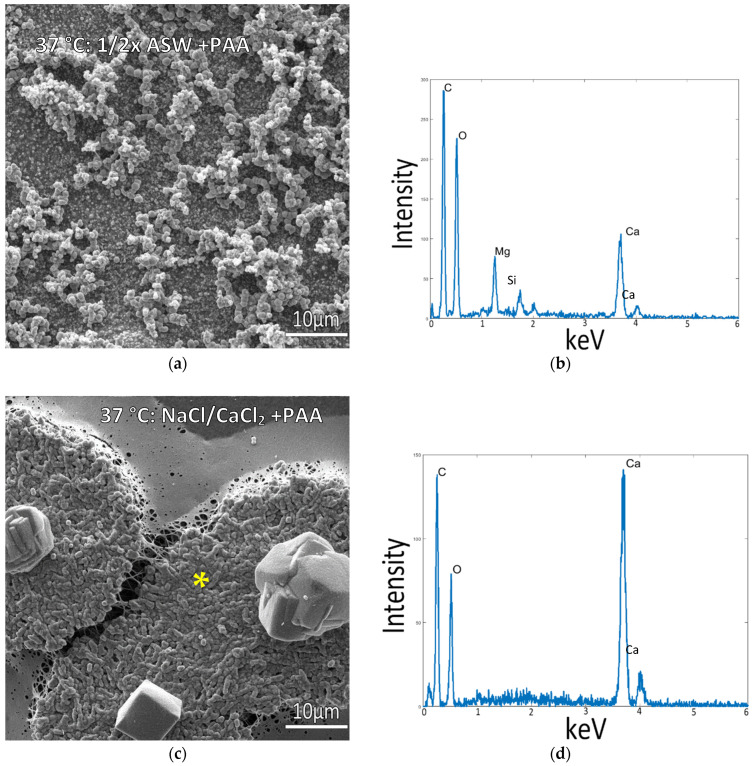
Collagen-coated glass coverslips incubated in mineralization solutions at 37 °C. (**a**) SEM image of collagen incubated in ½ ASW displays a large amount of mineral precipitates on the surface but a lack of infiltration into the collagen matrix. (**b**) EDS of sample (**a**) shows a moderate Ca peak from the granular precipitates. (**c**) SEM of collagen incubated in 150 mM NaCl + 10 mM CaCl_2_ solution (no Mg) displays raised portions where plump collagen fibrils are visible. (**d**) EDS taken from the raised patch of collagen surface in (**c**), marked by the asterisk, confirms the infiltration of calcium carbonate mineral into the fibrils.

Given the lack of intrafibrillar mineralization created by any of the room temperature conditions, we decided to examine the mammalian physiological temperature of 37 °C since the type 1 collagen used here is from a bovine source. Samples incubated in the standard ½ ASW + PAA mixes at 37 °C displayed a thick layer of micron-sized mineral aggregates coating the surface (Figure 4a). Even though the precipitates appear to be a mixture of both spherules and granular precipitates, the XRD analysis found only the aragonite phase (Supplementary Figure S1e). In contrast, samples incubated in solutions of NaCl and 10 mM CaCl_2_ containing PAA (without Mg) displayed raised patches, which were regions created by plumped-up collagen fibrils that were well infiltrated with minerals (Figure 4c). This is typical of intrafibrillar mineralization, where mineral displaces the water within collagen fibrils, keeping them from collapsing upon drying [29,38]. This is further evidenced by the large Ca peak in EDS in a region where no crystals or mineral coating are visually seen (Figure 4d), indicating the mineral is hidden within the fibrils. Agglomerates of the mineral were also present on most of these mineralized patches, but they appeared to be comprised of just a few distorted rhombs of calcite. The XRD pattern surprisingly shows a mix of aragonite and calcite phases, even though the solution did not contain Mg ions (Supplementary Figure S1f). Interestingly, these rhombs were nearly always in the center of the raised patches of mineralized collagen (Supplementary Figure S2 and Graphical Abstract images), appearing to be caused by a “spherulitic” mineral propagation pattern analogous to that recently reported by Macías-Sánchez et al. for mineralized collagenous tissues such as bone, dentin and turkey tendon [48]. Given the centralized rhomb seen in all of the raised patches, it appears the rhomb may serve as a collection site that accumulates the mineral precursor droplets. We have previously observed that CaCO_3_ PILP droplets preferentially adsorb onto existing mineral particles [49], sometimes accumulating into tower-like aggregates (unpublished observations), while other times forming bumpy coatings that sprout off mineral nanofibers [50]. In the work here with the underlying collagen matrix, it is unclear how such an accumulation site would transfer precursors to the surrounding matrix.

### 3.3. Mineralization at 30 °C

**Figure 5:**
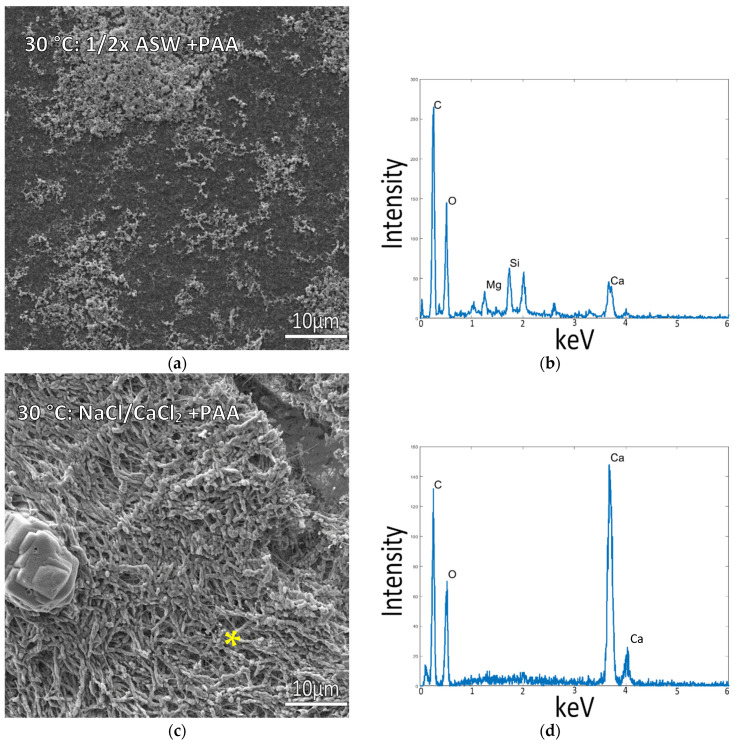
Collagen-coated glass coverslips incubated in mineralization solutions at 30 °C. (**a**) SEM of collagen incubated in ½ ASW displays a flat collagen surface with few mineral precipitates. (**b**) EDS of sample (**a**) shows a small Ca peak, presumably from the small surface precipitates. (**c**) Collagen incubated in 150 mM NaCl + 10 mM CaCl_2_ solution (no Mg) displays patches of raised collagen surface with visibly plump fibrils indicative of mineral infiltration into the matrix. (**d**) EDS Spectrum is taken from the raised patches of the collagen surface in (**c**), marked by the asterisk, with the large Ca peaks confirming mineral infiltration into the collagen fibrils.

As it became apparent that temperature had a strong influence on the ability to obtain intrafibrillar mineralization of the collagen, samples were also incubated at 30 °C to test if mineralization would still be viable under milder conditions that might improve the long-term viability of the marine invertebrate cells. Mineralization at 30 °C yielded similar results to the 37 °C mineralized samples. Incubating in ½ ASW resulted in poor mineralization with some mineral particles coating the surface (Figure 5a), while in NaCl and 10 mM CaCl_2_ samples (without Mg), raised patches of mineralized collagen were also present, with the CaCO_3_ mineral conforming to the shape of the collagen fibrils (Figure 5c,d, and left image of Graphical Abstract), similar to the 37 °C mineralized samples.

### 3.4. Influence of Calcium:Magnesium Ratio

**Figure 6:**
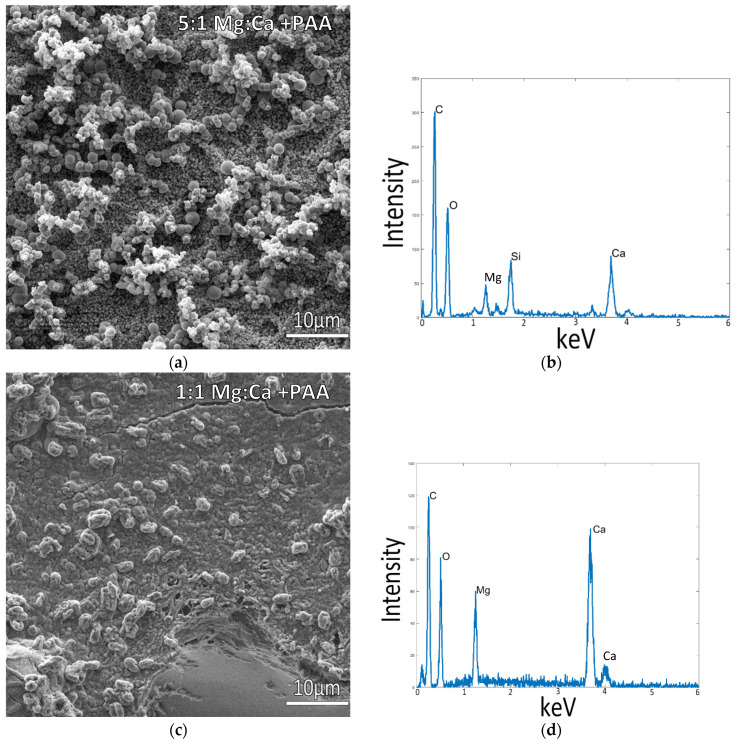
Collagen-coated glass coverslips incubated in mineralization solutions of variable Mg:Ca ratios, with PAA additive, at 37 °C. (**a**) SEM images of collagen incubated in 150 mM NaCl + 10 mM CaCl_2_ + 50 mM MgCl_2_ (5:1) displays a large amount of mineral precipitates, but the lack of visible fibrils indicates poor infiltration, as confirmed by the EDS spectrum in (**b**). (**c**) Collagen incubated in a 150 mM NaCl + 10 mM CaCl_2_ + 10 mM MgCl_2_ (1:1) displays a more cohesive mineral coating, but it appears to be on the raised collagen surface, where plump mineralized collagen fibrils are still visible. (**b**,**d**) display respective EDS spectra for the SEM images in (**a**,**c**), where spectrum (**d**) taken from the patches of raised surface of (**c**) indicates a large mineral presence on and within the fibrils of the collagen matrix.

As it was realized that both temperature and simplified mineral salt solutions had a dominant influence on the ability to obtain intrafibrillar mineralization of the collagen, we decided to retest some variables at 37 °C for two different levels of Mg. At a ratio of 5:1 Mg:Ca, the standard seawater ratio, the mineralization was disrupted, and there were no raised regions of mineralized collagen (Figure 6a), and mineral aggregates instead coated the flattened collagen surface. This also resulted in a change in the morphology of the extrafibrillar deposits relative to those seen in samples at lower temperatures (Figure 2a, Figure 3a, and Figure 5a), where they now appeared as more densely packed, rounded particulates that fully coated the collagen surface, along with some late-stage deposits that had grown into larger (roughly 2 microns) particles and spherules. Reducing the magnesium content to a ratio of 1:1 Mg:Ca, however, resulted in raised collagen patches similar to the previous mineralized samples without Mg, showing signs of mineral infiltration of the fibrils (Figure 6c,d). There appears to be so much mineral in this sample that it additionally led to a cementitious interfibrillar coating. The lumps protruding from the coating do not seem to be mineral crystals but rather appear to be lumpy protrusions of mineralized collagen. As a final control reaction, using the optimal mineralization conditions of 150 mM NaCl + 10 mM CaCl_2_ at 37 °C, but without the addition of PAA, no intrafibrillar mineralization was observed (Supplementary Figure S3).

## 4. Discussion

Collagen samples were mineralized in a variety of mineralization solutions and temperatures to assess the effect of various marine conditions on successful intrafibrillar mineralization of collagen organic matrices. Overall, the samples showed a trend of intrafibrillar collagen mineralization conforming to fibril shape at higher temperatures (30–37 °C) in solutions with low Mg content and poorer mineralization at room temperature or in solutions with higher Mg content in proportion to the Ca content.

The results show that both mineralization temperature and solution composition play a role in the effective mineralization of collagen in the presence of process-directing agents. The incorporation of charged polymers as process-directing agents is one factor that is essential to the mineralization of collagen fibers, shown by the absence of intrafibrillar mineralization in all samples without PAA, where the mineral instead deposited as a crust on the surface of the collagen film. Solution compositions containing magnesium at higher concentrations (including the 1/2x ASW), resulting in a layer of mineral on the surface of the respective matrices with poor intrafibrillar mineralization of collagen below the surface of the mineral particles and coatings (Figure 4a and Figure 6a). In solutions with CaCl_2_ and NaCl only, without Mg, and at temperatures of 30 °C and above, the mineral infiltrates into the collagen structure, coating and likely infiltrating the fibrils, as indicated by the SEM images and respective EDS spectra from raised collagen portions on these samples (Figure 4c,d and Figure 5c,d).

Inorganic additives have a significant impact on both depositions of calcium carbonate minerals and their interaction with organic matrices. Magnesium, in particular, has been shown to have an impact on mineralization behavior and mineral properties, noted in calcifying organisms such as crustaceans for its role in stabilizing ACP and ACC phases [51], and in vitro for its ability to enhance the PILP process and promote the deposition of amorphous CaCO_3_ films in synergy with polymer process-directing agents [51,52]. These in vitro studies have shown that at lower Mg:Ca ratios of up to 2, Mg is incorporated into lattice positions of the calcite structure, with differences in the short-range order around the Mg ions altering the morphology of the calcite mineral [53]. At higher Mg:Ca ratios >4, Mg has been shown to inhibit the nucleation of calcite, allowing for the nucleation of phases that are typically less stable, such as aragonite or ACC [53]. The inhibition of calcite growth and corresponding stabilization of amorphous phases lowers the amount of process-directing polymer needed for PILP formation and subsequent formation of non-equilibrium mineral morphologies [52]. However, in the presence of collagen, the incorporation of Mg here is seen to have negative effects on the mineral infiltration of collagen scaffolds. Disruptive effects of Mg on mineral infiltration were also observed by Ping et al. [39] during in vitro analysis of the driving force of amorphous mineral infiltration into collagen. Analysis of mineralized fibrils and ACC revealed that though there were many similarities between ACC and Mg-induced ACC, Mg changed the wettability and surface composition of the ACC, affecting its interaction with the fibrils and promoting attachment to the fibrils rather than infiltration as is thought to occur through capillary action [29]. The solution compositions used by Ping et al. were similar to those used in our current observations, with polymer and Mg-induced ACC at a ratio of 1:1 Mg:Ca but at lower concentrations (5 mM Mg and Ca). Solution compositions in our study with a Mg:Ca ratio of 1:1 resulted in infiltration of collagen with a different appearance than in the absence of Mg, where some individual mineralized fibrils are still visible, but a more continuous coating covers the surface of the collagen matrix as well. At larger Mg:Ca ratios, fibril mineralization does become completely disrupted, reverting to the appearance of the mineral crust covering unaffected collagen matrix, analogous to that seen in the mineralization solutions without added PAA. This indicates that Mg does alter the character of the amorphous phases generated through the PILP process, promoting deposition and attachment to but not infiltration of the collagen matrices at the higher Mg:Ca ratio. With respect to the debate on the mechanism of infiltration, the reduced efficiency of infiltration with the addition of Mg ions does not seem to support the hypothesis regarding intrafibrillar mineralization being based on the Gibbs–Donnan effect [54].

Another interesting observation is that the EDS Mg peak was smaller for the 5:1 Mg:Ca sample than the 1:1 Mg:Ca sample (Figure 6b,d, respectively), even though a far greater amount of Mg was in the solution. This is presumably due to differences in their precursor phases, given that the amorphous precursor pathway tends to entrap far more Mg ions within the lattice of its transformed calcite [52]. In addition, the 5:1 ratio prohibits intrafibrillar mineralization while the 1:1 ratio still allows for it, further suggesting that the non-classical behavior of the precursor phase is reduced at the 5:1 Mg:Ca ratio. Furthermore, Mg ions are well known to promote the aragonite phase, and although only calcite was formed from the ½ASW conditions on glass when PAA was added, it promoted some aragonite (Supplement Figure S1a vs. b). This was true for all the PAA conditions and seemed to be further promoted at the higher temperature. In fact, at the temperature of 37 °C, the ½ASW + PAA solution yielded pure aragonite (Figure 4a and Figure S1e). Unfortunately, this condition did not lead to intrafibrillar mineralization (because of the high Mg content). In contrast, the NaCl/CaCl_2_ + PAA solution, which did lead to intrafibrillar mineral, showed the presence of both aragonite and calcite (Figure 4c and Figure S1f). This was surprising given that this solution does not contain any Mg ions. Even more surprising is that those large extrafibrillar crystals, which are calcite rhombs, seemed to serve as “nucleation” centers for the rounded patches that became mineralized, so one might anticipate that those mineralized patches would also be calcite. We suspect this is the case and that the flat collagen matrix that was not well infiltrated with minerals might have been responsible for the aragonite signal, given the similar size/breadth of those peaks in all the PAA samples. Unfortunately, we could not separate those raised patches from the remaining flat collagen matrix to confirm. In conclusion, the synergy that we had expected from our prior work on CaCO_3_ film formation [52,55], where we found a combination of polymer + Mg ions enabled pure PILP films to be formed without aggregate byproducts, does not seem to hold true for intrafibrillar mineralization of collagen. This again highlights the complex interplay between insoluble organic matrices, mineral precursors, and the soluble organic and inorganic additives that induce them.

## 5. Conclusions

This work shows that a variety of intersecting conditions are needed for the successful mineralization of reconstituted collagen matrices. Insight into these biomineralization processes in vitro is useful in understanding the interplay between organic matrices and ambient solution conditions, as it will be needed for harnessing these interactions via cell-secreted IDPs to synthesize and mineralize matrices with non-native materials. The localized patches of the mineralized matrix are intriguing (Figure S2 and graphical abstract images) and illustrate the distinctly different processes of mineralization, where classical ion-by-ion growth generally leads to homogeneous deposits, while PILP droplets tend to accumulate into streams that then lead to patches of deposits [28]. A localization of mineralization is, in fact, what we anticipate seeing when the anemone cells secrete their biopolymers, which will likely induce and accumulate the PILP phase locally. It will be useful to have such a distinct visualization of successful mineralization. In future work, we plan to determine if collagen samples can be mineralized with polymer process-directing agents derived from natural biopolymers (IDPs) found in native biominerals, either isolated and added to the solution or provided by cell secretion into the matrices. The current tested conditions suggest a narrower range of temperatures and solution conditions may be needed, but it is possible that natural IDPs will be more effective than the simple PAA used in these preliminary studies, so this system provides a useful control reaction for comparison. The use of vibrational spectroscopy methods (FTIR or Raman) for the detection and analysis of ACC may also be useful in future work for the evaluation of potential precursor species involved in the successful mineralization of templates. In addition, this range of conditions can possibly be expanded using different substrates. For example, collagen derived from marine animals may be more stable at lower temperatures than mammalian collagen or exhibit more favorable interactions with mineral precursors at room temperature. Another option might be to use chitin matrices, which are the native organized matrices found in a variety of marine invertebrates. Continued testing of this mineralization process with different process-directing agents, substrates, and the incorporation of cells can provide more insight into the processes of cell-mediated biomineralization and the formation of new biomineralized structures.

## Figures and Tables

**Table 1:** Artificial seawater mixes and simplified compositions.

Component	Concentration (mM)			
	Instant Ocean Seawater Mix	1/2x ASW	NaCl + 10 mM Ca	NaCl + 10 mM Ca + 50 mM Mg
Chloride	544	272	160	160
Sodium	469	235	190	190
Sulfate	28	14	20	20
Magnesium	54	27	0	50
Potassium	10	5	10	10
Calcium	10	5	10	10
Carbonate/bicarbonate	3	2	0 *	0 *

* Carbonate/bicarbonate is added to mineralizing solutions via vapor diffusion.

## Data Availability

Not applicable.

## Supplementary Materials

The following supporting information can be downloaded at: https://www.mdpi.com/article/10.3390/biomimetics7030086/s1, Figure S1: XRD patterns of calcium carbonate mineral precipitated under the various mineralization conditions [56]; Figure S2. Lower magnification images of raised collagen patches on matrices with centralized rhombs; Figure S3. Collagen-coated glass incubated in optimal mineralization conditions but without the addition of PAA.
