# Supplementary material for: Assessment of Optimal Conditions for Marine Invertebrate Cell-Mediated Mineralization of Organic Matrices
Source: Biomimetics (Basel). 2022 Jun 26;7(3):86. doi: 10.3390/biomimetics7030086 (PMC9326593; doi:10.3390/biomimetics7030086)
Supplement: Supplementary file 1 [file biomimetics-07-00086-s001.zip › biomimetics-1780724-supplementary.pdf]

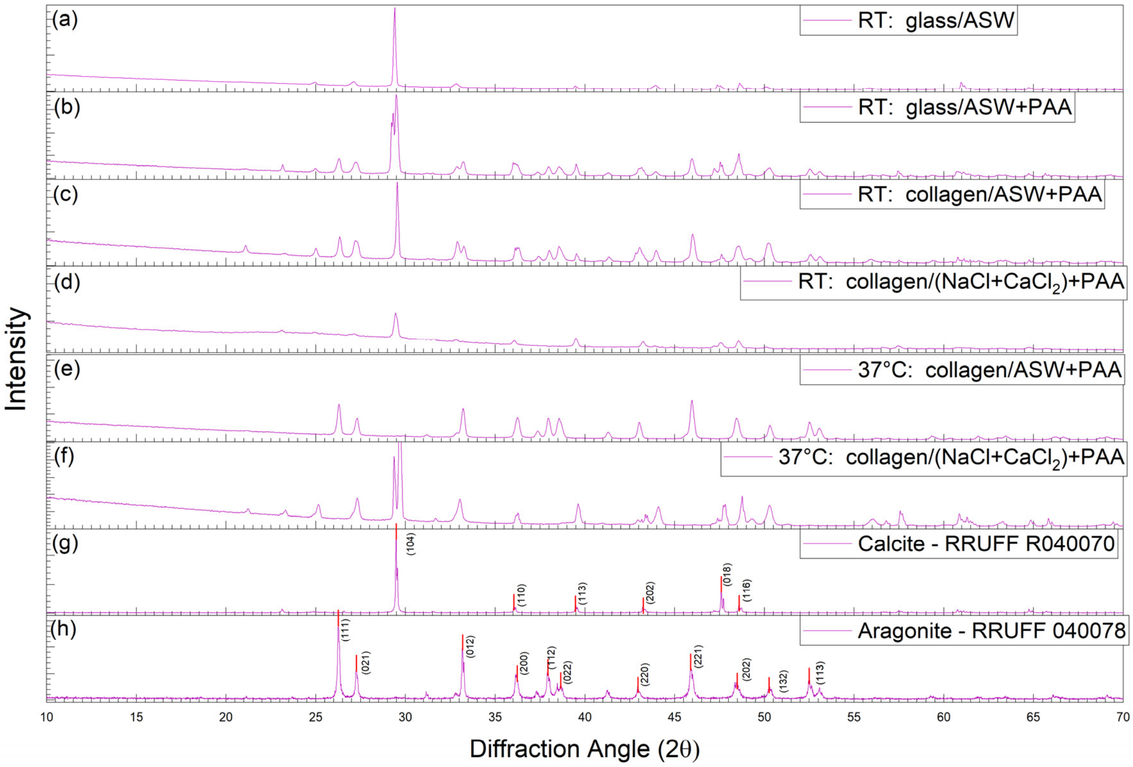

Figure S1. XRD patterns of calcium carbonate mineral precipitated under various conditions, with comparative reference patterns of calcite and aragonite (obtained from RRUFF sample data [1]) at the bottom. (a) On the glass slide, using 1/2x ASW without PAA as a process-directing agent, the mineral precipitates were mainly calcite; but in the presence of PAA, a mixture of calcite and aragonite crystals were present (b). (c) A similar mixture of peaks for the two phases occurred for the same conditions using the collagen substrate, although the 104 peak was more homogeneous. (d) At room temperature in solutions without Mg, mainly calcite was precipitated, with smaller peaks relative to the background due to the decreased mineral deposition. (e) At 37°C, the extrafibrillar mineral precipitated from 1/2x ASW + PAA was exclusively aragonite. (f) The mineral that infiltrated collagen fibrils in solutions without Mg at 37°C displayed peaks characteristic of calcite (with some aragonite), with a strongly split 104 peak.

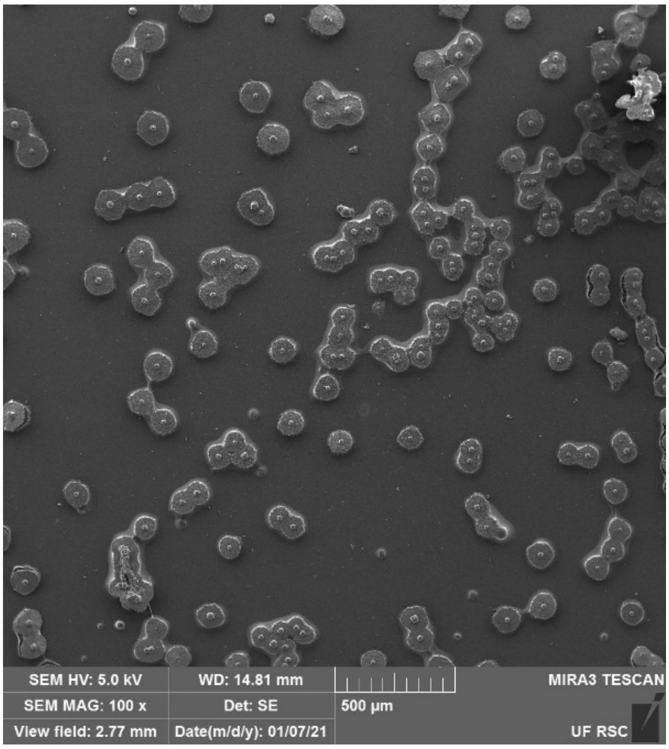

Figure S2. Lower magnification image showing many isolated raised patches on the collagen matrix that was incubated in 150 mM NaCl + 10 mM $CaCl_2$ solutions at 37°C. Most of the raised portions are circular and resemble spherulites, and they nearly always contain a calcite rhomb near the center of the patch, suggesting that the rhomb served as a "nucleation" or collection site where the mineral precursors accumulated.

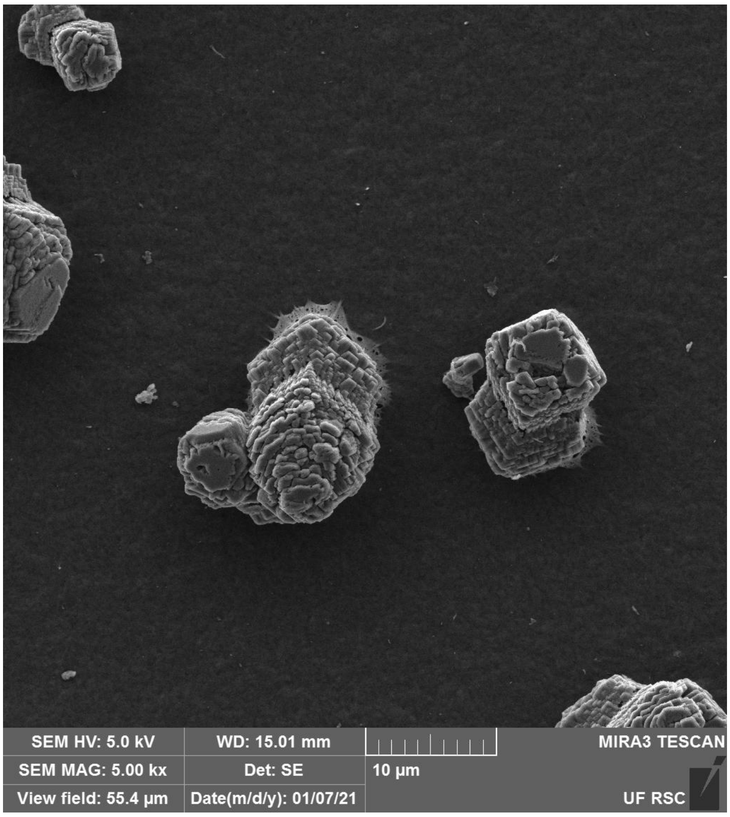

Figure S3. Collagen-coated glass cover slips incubated in the optimal mineralization conditions of 150 mM NaCl + 10 mM $CaCl_2$ at 37°C, but without the addition of PAA, showing only calcite crystals on a flat collagen matrix. The rhombs are rough and severely distorted under these conditions.

[1] Antao SM. Temperature dependence of the structural parameters in the transformation of aragonite to calcite, as determined from in situ synchrotron powder x-ray-diffraction data. Can Mineral 2010;48:1225–36.
